# A novel polyamine-type starch/glycidyl methacrylate copolymer for adsorption of Pb(II), Cu(II), Cd(II) and Cr(III) ions from aqueous solutions

**DOI:** 10.1098/rsos.180281

**Published:** 2018-06-06

**Authors:** Youning Chen, Wei Zhao, Huan Wang, Xiaohua Meng, Linjie Zhang

**Affiliations:** 1 College of Chemistry and Chemical Engineering, Xianyang Normal College, Xianyang 712000, People's Republic of China; 2 State Key Laboratory of Integrated Services Networks, Xidian University, Xi'an 710071, People's Republic of China

**Keywords:** amino modified starch, adsorption, heavy metal ion

## Abstract

A novel polyamine-type starch/glycidyl methacrylate (GMA) copolymer with a high capacity for the adsorption of heavy metal ions was prepared via graft copolymerization of GMA and corn starch and a subsequent amination reaction between amino group of diethylenetriamine and epoxy group in GMA. The copolymers were characterized by Fourier transform infrared spectrometry, X-ray diffraction and scanning electron microscopy, and adsorption properties on modified starch of Cu(II), Pb(II), Cd(II) and Cr(III) were studied. By analysing the relationship between adsorption capacity and pH, adsorption isotherms and adsorption kinetics, it is proved that the adsorption of the four metal ions is mainly based on the chemical adsorption of coordination. The maximum adsorption capacities of the copolymer were up to 2.33, 1.25, 0.83 and 0.56 mmol g^−1^ for Cu(II), Pb(II), Cd(II) and Cr(III), respectively. The adsorption of the four concerned metal ions was hardly affected by common coexisting ions such as Na(I), K(I), Ca(II) and Mg(II), whereas it was slightly decreased when Fe(II) and Zn(II) coexisted in the solution, which illustrates the selective adsorption of Cu(II), Pb(II), Cd(II) and Cr(III) from wastewater. After 10 cycles of adsorption–desorption experiments, there was no significant change in the adsorption capacity, indicating that the polyamine-type starch/GMA copolymer has high adsorption capacity and good reusability.

## 1. Introduction

With the rapid development of modern industrial technology, the problem of heavy metal pollution in water is worsening day by day [1,2]. Heavy metal ions are discharged to the natural environment mainly through smelting, electroplating and other industrial wastes, and municipal wastewater. Then through the enrichment of the food chain, they enter plant and animal bodies, which in turn threaten human health. The traditional methods of heavy metal wastewater treatment include chemical precipitation, ion exchange, reverse osmosis, membrane technology, distillation, electrochemical treatment and electrodialysis [3–6]. These methods have good results to a certain extent; however, the problems of high cost, low removal rate and easy secondary pollution are common. Among them, the adsorption method has the advantages of low cost and simple process, and is an extremely effective method for removing heavy metals in wastewater [7–10].

In recent years, cheaper and more effective adsorbents have drawn much attention. Starch-based heavy metal adsorbents, with the advantages of a wide range of raw materials, low cost, non-toxic, harmless, easily biodegradable and excellent adsorption effects, get more and more attention and application [11–13]. At present, graft copolymerization of vinyl monomer is the main method of starch modification. Grafted starch copolymers, such as acrylamide, acrylonitrile, acrylic ester and acrylic acid, have been reported as adsorbents for heavy metal wastewater [14–17]. However, the effective functional group content is low, and the adsorption effect is not very satisfactory. There are fewer reports on further functional modification of starch graft copolymers to obtain adsorbents with high nitrogen content and their applications for heavy metal wastewater treatment.

In this paper, glycidyl methacrylate (GMA) and starch were graft-copolymerized, and then the polyamine-type starch/GMA copolymer (PAMS) was prepared through the cycloaddition reaction between the amine groups in diethylenetriamine and the epoxy groups in GMA. The adsorption properties of Cu(II), Pb(II), Cd(II) and Cr(III) in solution were also investigated. It provides some theoretical basis for the application of PAMS in the treatment of heavy metal wastewater.

## 2. Experimental

### 2.1. Materials

Corn starch (Xi'an Chemical Reagent Factory, China), GMA (CP, Shanghai Aladdin), diethylenetriamine (AR, Shanghai Aladdin), potassium persulfate (AR, Tianjin Fuchen Chemical Reagent Factory), OP-10 (AR, Tianjin Fuchen Chemical Reagent Factory), acetone (CP, Luoyang Chemical Reagent Factory), ethanol (CP, Luoyang Chemical Reagent Factory), CuSO_4_·5H_2_O (AR, Tianjin Fuchen Chemical Reagent Factory), Pb(NO_3_)_2_ (AR, Tianjin Fuchen Chemical Reagent Factory), Cr(NO_3_)_3_·9H_2_O (AR, Tianjin Fuchen Chemical Reagent Factory), Cd(NO_3_)_2_·4H_2_O (AR, Tianjin Fuchen Chemical Reagent Factory).

### 2.2. Preparation of starch/glycidyl methacrylate copolymer

Five grams of starch and distilled water were placed into a four-necked flask equipped with a thermometer and nitrogen protection device. After stirring for a period of time, an emulsifier, 10.0 g GMA and initiator (potassium persulfate) were added. The reaction was performed at 60°C under stirring and a nitrogen atmosphere. The product was poured into anhydrous ethanol in order to demulsify, soaked at room temperature for 30 min to remove unreacted monomer, and, after suction filtration, dried at 60°C to obtain crude graft copolymer. Homopolymer was then removed via Soxhlet acetone extraction for 24 h.

### 2.3. Preparation of polyamine-type starch/glycidyl methacrylate copolymer

Two grams of starch/GMA copolymer (St-g-GMA) and 30 ml of diethylenetriamine were added into a 250 ml three-necked flask equipped with an electric stirrer, a thermometer and a reflux condenser, and the mixture was refluxed with stirring at 90° C for 10 h. The reactant was precipitated with anhydrous ethanol, washed with distilled water until neutral, suction filtered, and dried at 70°C.

### 2.4. Characterization of the chemical composition

Chemical composition information was analysed using a Tensor 27 FT-IR spectrophotometer (Bruker Company, Germany) and X-ray diffractometer (XD-3, Beijing general analysis of General Instrument Co., Ltd, China).

### 2.5. Batch adsorption experiment

The adsorption performance on PAMS of Cu(II), Pb(II), Cd(II) and Cr(III) was studied. Under the conditions of no competition, the PAMS was placed into the solution containing one kind of metal ion for adsorption.

#### 2.5.1. Effect of pH

One gram of PAMS was added to a series of 5 mmol l^−1^ solutions (100 ml) containing Cu(II), Pb(II), Cd(II) and Cr(III) with different initial pH values. The pH value ranged from 1.0 to 7.0. The mixtures were shaken at 200 r.p.m. for 12 h at 25°C. After the adsorption was completed, the copolymer was separated by membrane filtration of 0.2 µm, the concentration of metal ions in the filtrate was measured by atomic absorption spectroscopy (AAS), and the adsorption amount of the copolymer was calculated by the following equation:

2.1 $$Q_{e}=\frac{(C_{0}-C_{e})V}{W},$$

where *Q*_e_ is the equilibrium adsorption capacity (mmol g^−1^), *C*_0_ and *C*_e_ are the initial and equilibrium metal ion concentrations (mmol l^−1^), respectively, *V* is the solution volume (l), and *W* is the mass of the dried copolymer (g).

#### 2.5.2. Adsorption isotherm

Cu(II), Pb(II), Cd(II) and Cr(III) stock solutions were adjusted to pH 5.0 and then diluted with NaAc-HAc buffer solution to obtain solutions of different concentrations (0.5–5.0 mmol l^−1^). In total, 0.100 g of PAMS was placed in a series of flasks containing different initial concentrations of each metal ion. Each flask was agitated at 200 r.p.m. at 25°C for 12 h. The adsorption isotherm of each metal ion was obtained by plotting *Q*_e_ against *C*_e_.

#### 2.5.3. Adsorption kinetics

To a series of 0.1 g PAMS, 100.00 ml of 5.0 mmol l^−1^ Cu(II), Pb(II), Cd(II) and Cr(III) working solutions were added separately in vessels, and then the mixtures were shaken on a shaker at 25°C. Aliquots of 1.00 ml solution were taken at different time intervals, and the concentration variations of metal ions were analysed using AAS. The kinetic curve was obtained by plot of *Q*_e_ versus adsorption time.

### 2.6. Adsorption selectivity

The adsorption selectivity of the copolymer was studied under the condition of competition; a binary system consisting of 0.1 g PAMS and 100 ml solution containing Cu(II), Pb(II), Cd(II) and Cr(III) (initial concentration 5.0 mmol l^−1^, pH 5.0) and coexisting ions (Na(I), K(I), Ca(II), Mg(II), Fe(III) and Zn(II), 10.0 mmol l^−1^) were added into a series of 250 ml conical flasks, then the mixtures were shaken on a shaker at 25°C.

### 2.7. Competitive adsorption

In total, 0.100 g PAMS was placed in a series of flasks containing 100 ml of the mixture solution including two, three and four metal ions (the concentration of each metal ion was 5.0 mmol l^−1^).

### 2.8. Recycling experiments

Cu(II)-, Pb(II)-, Cd(II)- and Cr(III)-loaded PAMS were regenerated with 0.1 mol l^−1^ HNO_3_ solution and then collected by filtration, washed with distilled water and reused in the next cycle of adsorption experiments.

## 3. Results and discussion

### 3.1. Preparation of polyamine-type starch/glycidyl methacrylate copolymer

**Figure 1.**
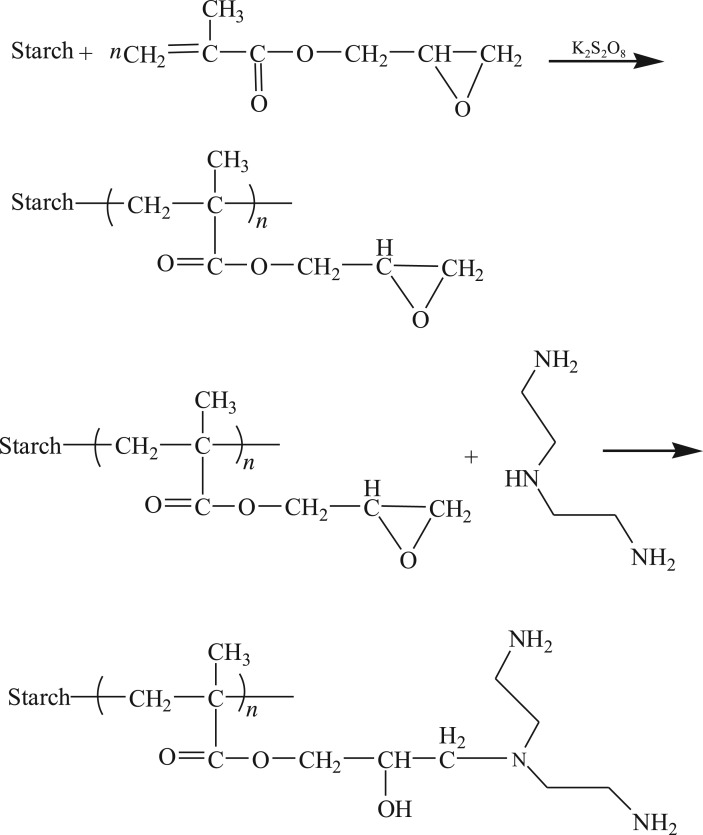
Synthetic route for the preparation of PAMS.

PAMS was prepared via a two-step method, shown in figure 1. In the first step, GMA was graft-copolymerized with starch to introduce epoxy groups.

**Figure 2.**
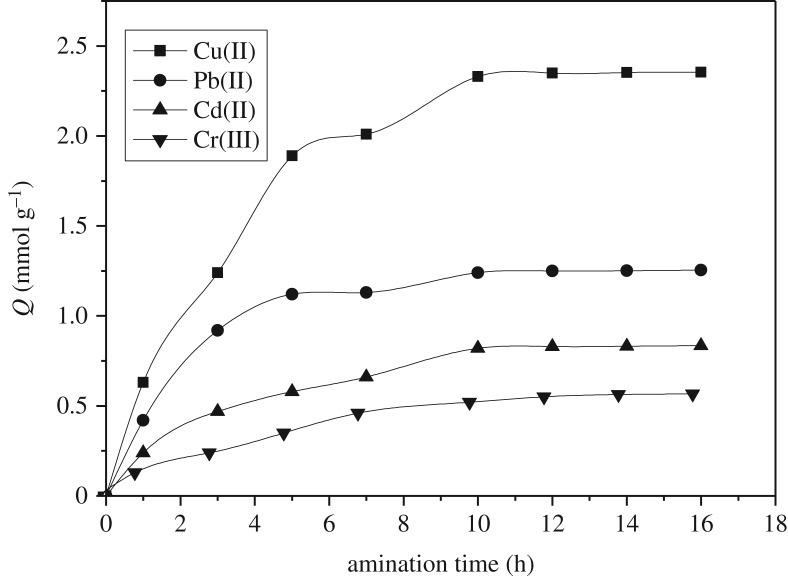
Effects of the amination reaction time on the sorption capacity.

In the second step, PAMS was obtained through the amination reaction between amino group of diethylenetriamine and epoxy group in GMA. Figure 2 shows the effect of open-loop times on the adsorption capacities of Cu(II), Pb(II), Cd(II) and Cr(III). Within 7 h of ring-opening reaction, the adsorption capacity increased rapidly, and the adsorption equilibrium was reached in about 10 h. As amine groups are primarily responsible for metal ion adsorption, an increase in adsorption capacity indicates that more amine groups are incorporated into the copolymer by reaction with the epoxy groups in GMA. Therefore, the open-loop reaction time was fixed at 10 h to prepare PAMS required for the subsequent adsorption experiments.

### 3.2. Characterization of the chemical composition

The chemical compositions of starch, St-g-GMA and PAMS were characterized by scanning electron microscopy (SEM), Fourier transform infrared (FTIR) spectroscopy and X-ray diffraction (XRD).

**Figure 3.**
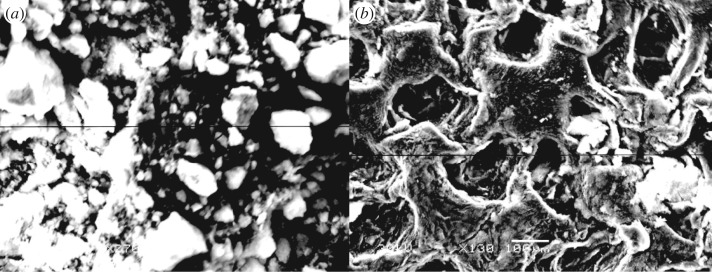
SEM micrographs of (*a*) starch and (*b*) PAMS.

As can be seen from SEM (figure 3), the starch granules are spherical, with smooth surface, compact structure and clear interface. Compared with starch, the surface of PAMS is rougher and has higher porosity with slit, loose structure. The shape and structure of PAMS are more conducive to the adsorption process.

**Figure 4.**
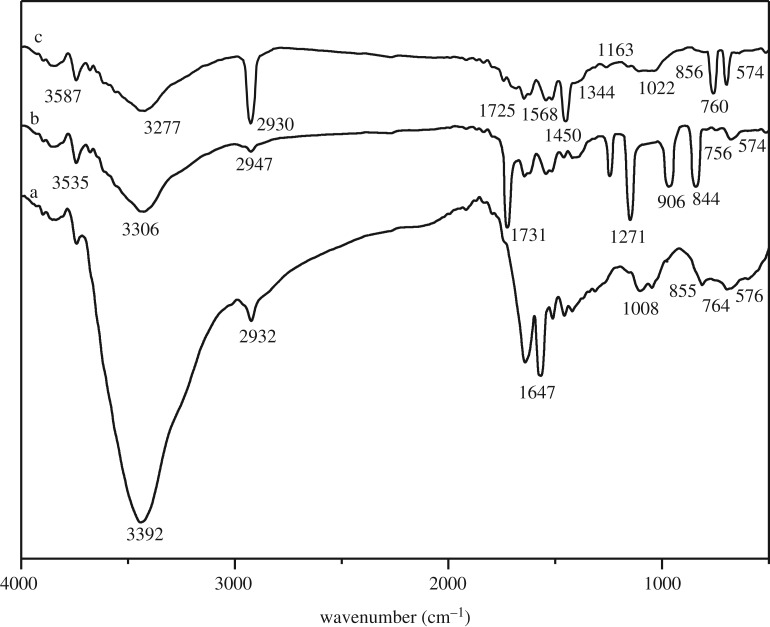
FTIR spectra of (*a*) starch, (*b*) St-g-GMA and (*c*) PAMS.

Typical FTIR spectra are depicted in figure 4. In the curves of starch, St-g-GMA and PAMS, there is a strong absorption peak of –OH stretching vibration of starch near 3392 cm^−1^, a significant saturated C–H stretching vibration peak near 2932 cm^−1^ and a C–O stretching vibration peak near 1008 cm^−1^, and starch characteristic absorption peaks at 576, 764 and 855 cm^−1^. In figure 4*b*, it was also found that the strong peak at 3535 cm^−1^, due to intermolecular –OH association, suggests the occurrence of cross-linking reaction. The characteristic peak of carbonyl group appeared at 1731 cm^−1^, and the characteristic peaks of epoxy group appeared at 1271, 906 and 844 cm^−1^, which indicated the successful graft copolymerization of starch with GMA. In figure 4*c*, the peak at 3277 cm^−1^ was caused by the superimposition of –OH stretching vibration absorption peak and N–H stretching vibration absorption peak, and the peak at 1568 cm^−1^ corresponding to the bending vibration of –NH_2_ indicates that PAMS contains amino group.

**Figure 5.**
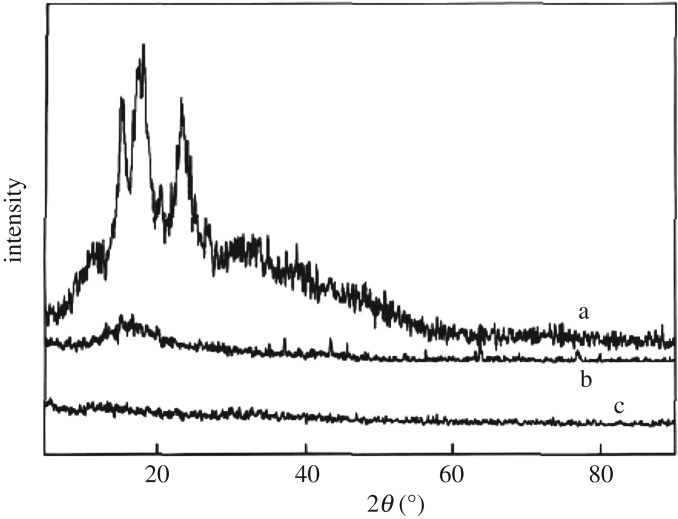
X-ray diffraction patterns of (*a*) starch, (*b*) PAMS and (*c*) PAMS--Cu(II).

In XRD patterns (figure 5), there are strong characteristic diffraction peaks at 16.3°, 18.24°, 22.98° (figure 5*a*), indicating that there is a certain crystalline structure in starch. In figure 5*b*, the diffraction peak appears slightly at 18.5°; the XRD peak of PAMS exhibits a big difference from starch. In the process of grafting and functional modification, the original state of aggregation has changed; the crystalline structure of starch particles has been severely damaged, reducing its crystallinity. After the adsorption of Cu(II), the intensity of the diffraction peak near 18.5° became weaker, indicating the degree of crystallinity is further reduced. Cu(II) interferes with the movement of the molecular chain after entering the interior of the PAMS, destroying the regular arrangement of molecular chains. In addition, the coordination of Cu(II) with –NH_2_ on PAMS will destroy the intermolecular (internal) hydrogen bonds, resulting in the destruction of the crystalline region of PAMS and the decrease of crystallinity.

### 3.3. Batch adsorption property of heavy metal ions

#### 3.3.1. Effect of pH on adsorption

**Figure 6.**
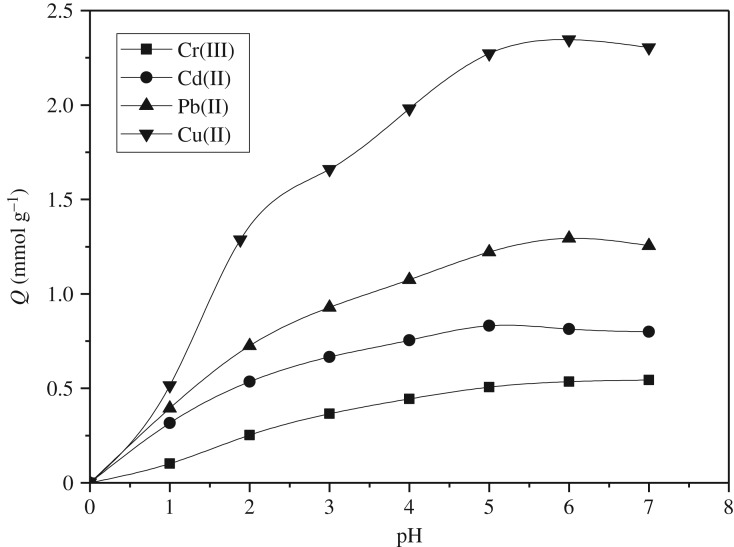
Effect of pH on the adsorption of PAMS for Cu(II), Pb(II), Cd(II) and Cr(III) (initial concentration: 5 mmol l^−1^; 25°C; adsorbent dose: 0.1 g).

Figure 6 shows the effect of pH on adsorption capacity. To avoid the formation of hydroxide precipitate, the pH values of Cu(II), Pb(II), Cd(II) and Cr(III) solutions were controlled below 7.0. It can be seen from figure 6 that the adsorption capacity of Cu(II), Pb(II), Cd(II) and Cr(III) increased with the increase of the pH value. When the pH value is less than 2.0, PAMS had almost no adsorption of Cu(II), Pb(II), Cd(II) and Cr(III). Because of the lower pH, the amine groups become protonated and the cations cannot approach the adsorption sites due to electrostatic repulsion. With the increase of the pH value, some of the amine groups in PAMS could adsorb Cu(II), Pb(II), Cd(II) and Cr(III) via chelation. The low adsorption capacity at lower pH indicated that the copolymer could be regenerated in acidic solution. The maximum adsorption capacity for Pb(II), Cu(II), Cd(II) and Cr(III) was found at pH 4.0–7.0. To exclude the possibility of the hydrolysis of metal ions, pH 5.0 was selected as the optimum pH for the subsequent adsorption experiments.

#### 3.3.2. Adsorption isotherm

**Figure 7.**
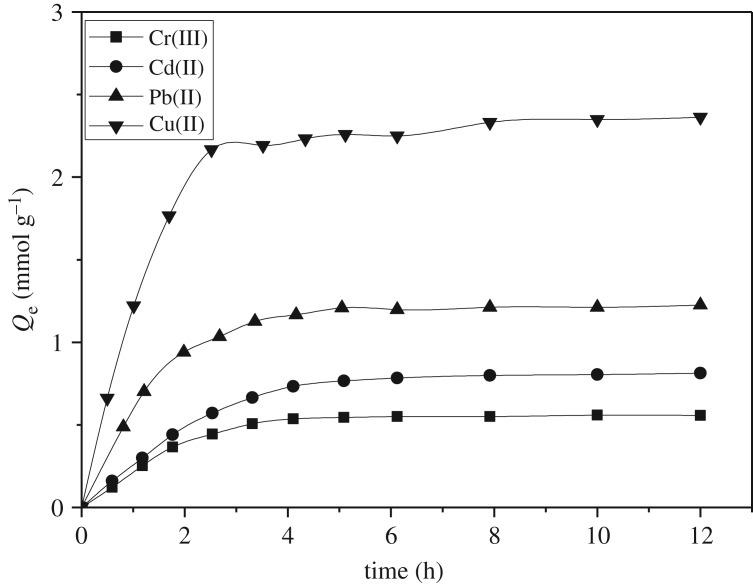
Adsorption kinetics of PAMS for Cu(II), Pb(II), Cd(II) and Cr(III) (initial concentration: 5 mmol l^−1^; 25°C; pH 5.0; adsorbent dose: 0.1 g)

Adsorption isotherm is often used to measure the adsorption performance of an adsorbent for heavy metal ions. The adsorption isotherms of PAMS for Cu(II), Pb(II), Cd(II) and Cr(III) at 25°C are presented in figure 7. The experimental data of adsorption were analysed by Langmuir model (3.1) and Freundlich model (3.2). The results are shown in table 1:

3.1 $$\frac{C_{e}}{Q_{e}}=\frac{C_{e}}{Q_{0}}+\frac{1}{Q_{0}b}\;\;b=(K_{c}-1)\times \frac{M}{\rho }$$

and

3.2 $$\ln Q_{e}=\ln K_{F}+\frac{1}{n}\ln C_{e},$$

where *Q*_e_ is the adsorption capacity, mmol g^−1^; *C*_e_ is the equilibrium concentration of metal ions, mmol l^−1^; *Q*_0_ is the saturated adsorption capacity, mmol g^−1^; *M* and *ρ* are the molar mass (g mol^−1^) and density (g l^−1^) of the solvent water; *K*_F_ is an empirical parameter; *n* is the Freundlich constant; and *K*_F_ is the binding energy constant reflecting the affinity of the adsorbents to metal ions.

**Table 1.** Langmuir and Freundlich constants for Cu(II), Pb(II), Cd(II) and Cr(III) adsorption on PAMS at 25°C.

	Langmuir parameters			Freundlich parameters		
metal ions	Q_0_ (mmol g^−1^)	*K*_c_	$R_{L}^{2}$	*K*_F_	1/*n*	$R_{F}^{2}$
Cu(II)	2.33	60.32	0.99544	0.352	0.51325	0.92543
Pb(II)	1.25	72.24	0.98563	0.521	0.54781	0.94621
Cd(II)	0.83	55.78	0.99243	0.798	0.59658	0.93762
Cr(III)	0.56	61.5	0.98687	1.224	0.5623	0.96854

It can be seen that the linear correlation coefficients obtained by the Langmuir fitting are better than those obtained by the fitting with Freundlich, indicating that the adsorption of Cu(II), Pb(II), Cd(II) and Cr(III) belongs to Langmuir monolayer adsorption model, which is the inevitable result of chemical adsorption process. According to the results of Langmuir fitting, the Langmuir constants *b* of Cu(II), Pb(II), Cd(II) and Cr(III) are 1.07 l mmol^−1^, 1.28 l mmol^−1^, 0.99 l mmol^−1^ and 1.09 l mmol^−1^, respectively.

According to the Langmuir fitting results, the maximum adsorption capacity (*Q*_m_) was 2.33 mmol g^−1^ for Cu(II), 1.25 mmol g^−1^ for Pb(II), 0.83 mmol g^−1^ for Cd(II) and 0.56 mmol g^−1^ for Cr(III). Compared with other absorbents (table 2), apparently PAMS had a higher adsorptive capacity for Cu(II), Pb(II), Cd(II) and Cr(III).

**Table 2.** Comparison of Cu(II), Pb(II), Cd(II) and Cr(III) adsorption on PAMS with other adsorbents.

	adsorption capacities (mmol g^−1^)				
adsorbents	Cu(II)	Pb(II)	Cd(II)	Cr(III)	reference
oxidized starch nanoparticles	1.27	0.46	—	—	[18]
cross-linked carboxymethyl starch	—	0.27	0.32	—	[19]
magnetic hydrogel beads based on poly(vinyl alcohol)/carboxymethyl starch-g-poly(vinyl imidazole)	1.31	0.31	0.47	—	[20]
semi-interpenetrating polymer network cryogels based on polyacrylamide and anionically modified potato starch	0.64	—	0.17	—	[21]
dithiocarbamate-modified GMA starch	0.32	—	0.25	—	[22]
porous starch xanthate	—	0.53	—	—	[23]
phosphorylation of chitosan/2-hydroxyethyl methacrylate interpenetrating polymer	1.04	—	—	—	[24]
rectorite with carbon layers and trisodium trimetaphosphate	—	1.247	—	—	[25]
peat	0.13	0.19	—	0.25	[26]
polyamine-type starch/GMA copolymer	2.33	1.25	0.83	0.56	this work

#### 3.3.3. Adsorption kinetics

**Figure 8.**
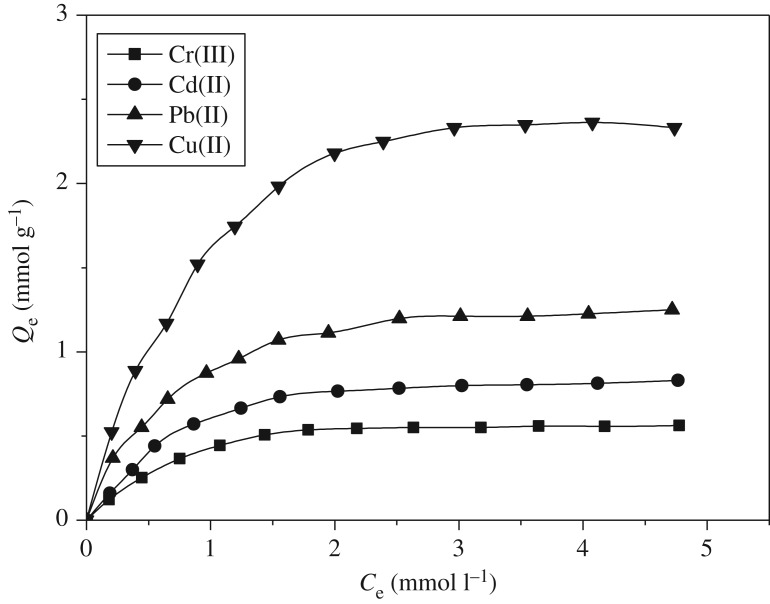
Adsorption isotherms of PAMS for Cu(II), Pb(II), Cd(II) and Cr(III) at 25°C (pH 5.0; contact time: 12 h; adsorbent dose: 0.1 g)

Adsorption kinetics describes the adsorption rate of metal ions, which is one of the important factors to study the PAMS adsorption performance. As shown in figure 8, the adsorption capacity of the four metal ions increased significantly within 60 min, and gradually reached the adsorption equilibrium.

The pseudo second-order kinetic equation was employed to analyse the adsorption kinetics of the four ions. The pseudo second-order equation is as follows:

3.3 $$\frac{t}{Q_{t}}=\frac{1}{kQ_{e}^{2}}+\frac{1}{Q_{e}}t,$$

where *t* is the adsorption time (min); *k* is the adsorption rate constant (min^−1^); and *Q*_*t*_ and *Q*_e_ are the adsorption amount at given time *t* and equilibrium time, respectively.

Equation (3.3) is used for the curve fitting of the adsorption kinetic data, and plotting *t*/*Q*_*t*_ against *t* generates a straight line. Good linearity was found for the four metal ions with correlation coefficients (*R*^2^) greater than 0.996, indicating that the adsorption of Cu(II), Pb(II), Cd(II) and Cr(III) by PAMS accorded with the quasi-second-order kinetic model, and the adsorption process is the chemisorption caused by the electrostatic interaction or coordination between adsorbent and adsorbate.

The adsorption rate constants obtained from the slopes and intercept of the plots were 2.238, 0.776, 1.455 and 0.834 mmol^−1^ h^−1^ for Cu(II), Pb(II), Cd(II) and Cr(III), respectively.

### 3.4. Adsorption selectivity

The adsorption selectivity is an indispensable factor for evaluating the properties of an adsorbent. In wastewater, the common ions coexisting with Pb(II), Cu(II), Cd(II) or Cr(III) are mainly Na(I), K(I), Ca(II), Mg(II), Fe(III) and Zn(II), which are biologically essential metals. Therefore, we studied the effect of coexistence of these ions on the adsorption of Cu(II), Pb(II), Cd(II) and Cr(III). As shown in table 3, the influences of Na(I), K(I), Ca(II) and Mg(II) on the adsorption of Cu(II), Pb(II), Cd(II) and Cr(III) can be ignored, but Fe(III) and Zn(II) displayed a minor influence. The HSAB principle (the theory of hard and soft acids and bases) can be used to explain this phenomenon. The amine group has the property of a soft base. Accordingly, compared to Na(I), K(I), Ca(II) and Mg(II) (hard acids), the metals Fe(III) and Zn(II), being borderline acids, should readily bind with the soft base and can thus interfere the adsorption of Cu(II), Pb(II), Cd(II) and Cr(III). The results indicate that PAMS can selectively adsorb Cu(II), Pb(II), Cd(II) and Cr(III) from wastewater.

**Table 3.** Effect of coexisting metal ions on the adsorption capacity of Pb(II), Cu(II), Cd(II) and Cr(III) (pH 5.0; contact time: 12 h; adsorbent dose: 0.1 g).

	selective coefficients			
coexisting ions	Cu(II)	Pb(II)	Cd(II)	Cr(III)
K^+^	∞	∞	∞	∞
Na^+^	∞	∞	∞	∞
Ca^2+^	89.6	66.5	51.8	43.5
Mg^2+^	77.4	43.5	34.1	28.5
Fe^3+^	31.2	24.3	21.2	18.2
Zn^2+^	20.5	15.8	14.6	11.8

### 3.5. Competitive adsorption

The competitive adsorption among Cu(II), Pb(II), Cd(II) and Cr(III) was also examined in a binary system, ternary system and quaternary system. The initial concentration of each metal ion in the mixed solution was 5.0 mmol l^−1^. As shown in table 4, in a binary system and ternary system, the presence of Pb(II), Cd(II) and Cr(III) had little effect on the adsorption of Cu(II). When four metals were present, PAMS adsorbed the heavy metals in the following order: Cu(II) > Pb(II) > Cd(II) > Cr(III). This affinity order was the same as in the single metal adsorption studies. This is probably because the hydrolysis constants of Cd(II) (pK = 10.1) and Cr(III) (pK = 9.7) are higher than that of Cu(II) (pK = 8.0). At the same pH, the proportions of CdOH^+^ and CrOH^2+^ are much lower than that of CuOH^+^, and the hydroxyl metal ions are more easily adsorbed. In addition, from the viewpoint of ion hydration radius, Cd(II) and Cr(III) are larger, which is unfavourable for adsorption. When there is a competitive ion with strong adsorption capacity, the adsorption capacities for Cd(II) and Cr(III) are reduced.

**Table 4.** Competitive adsorptions among Pb(II), Cu(II), Cd(II) and Cr(III) in binary, ternary and quaternary systems.

	removal efficiency (%)			
metal system	Cu(II)	Pb(II)	Cd(II)	Cr(III)
Cu(II)–Pb(II)	88.5	64.3	—	—
Cu(II)–Cd(II)	89.4	—	34.5	
Cu(II)–Cr(III)	90.6	—	—	32.6
Pb(II)–Cd(II)	—	67.8	37.9	—
Pb(II)–Cr(III)	—	69.4	—	35.8
Cd(II)–Cr(III)	—	—	40.8	37.9
Cu(II)–Pb(II)–Cd(II)	87.6	60.1	30.2	—
Cu(II)–Pb(II)–Cr(III)	88.2	60.6	—	26.5
Cu(II)–Cd(II)–Cr(III)	89.5	—	32.6	27.8
Pb(II)–Cd(II)–Cr(III)	—	64.2	34.1	29.8
Cu(II)–Pb(II)–Cd(II)–Cr(III)	85.4	52.3	34.2	25.2

### 3.6. Adsorption mechanisms

**Figure 9.**
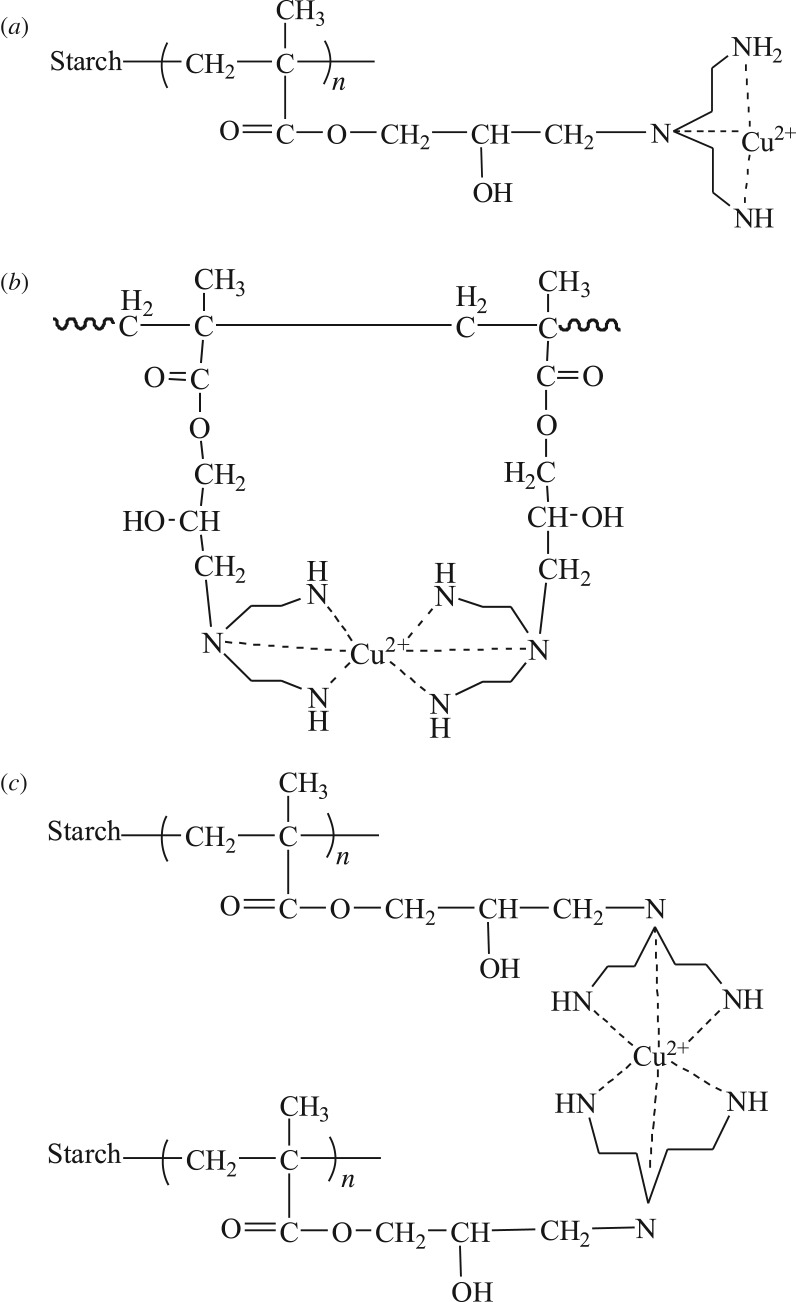
Adsorption mechanism of PAMS for Cu(II): coordinated with (*a*) three and (*b*) six amine groups in one polymer chain and (*c*) multiple amine groups in two adjacent polymer chains.

Amino groups in PAMS were demonstrated to act as adsorption sites for heavy metal ions. One metal ion may be surrounded by three amine groups (figure 9*a*) or six adjacent amine groups (figure 9*b*) in one polymer chain, and multiple amine groups in two adjacent polymer chains (figure 9*c*). Therefore, a metal ion can coordinate with three or six amine groups through coordination (with Cu(II) as the example).

### 3.7. Reusability of polyamine-type starch/glycidyl methacrylate copolymer

Cu(II)-, Pb(II)-, Cd(II)- and Cr(III)-loaded PAMS were placed in 1.0 mol l^−1^ HNO_3_ aqueous solution, and the amount of metal ions released in 2 h was determined. Desorption efficiency was found to be generally high (more than 97%). To investigate the reusability properties of PAMS, an adsorption–desorption cycle of metal ions was repeated ten times. As shown in table 5, after ten cycles of adsorption–desorption, the adsorption capacity of PAMS is decreased by 3.86% for Cu(II), 6.40% for Pb(II), 7.22% for Cd(II) and 7.14% for Cr(III), indicating that the adsorption capacity of PAMS did not change significantly after multiple adsorption–desorption cycles.

**Table 5.** Adsorption capacity of the resin after ten adsorption–desorption cycles.

	adsorption amount (mmol g^−1^)			
cycle	Cu(II)	Pb(II)	Cd(II)	Cr(III)
1	2.33	1.25	0.83	0.56
2	2.31	1.21	0.80	0.55
3	2.28	1.23	0.79	0.56
4	2.29	1.19	0.79	0.54
5	2.25	1.15	0.80	0.53
6	2.22	1.17	0.78	0.53
7	2.26	1.21	0.76	0.52
8	2.31	1.20	0.79	0.53
9	2.28	1.18	0.78	0.53
10	2.24	1.17	0.77	0.52

## 4. Conclusion

In this work, a novel PAMS with a high content of nitrogen was prepared through graft copolymerization and epoxy ring-opening reaction. SEM images showed that PAMS with a rough surface, a gap, loose structure and higher porosity was conducive to the adsorption process. FTIR confirmed the successful grafting of St-g-GMA and the formation of PAMS. XRD analysis showed that the crystallinity of starch, PAMS and Cu(II)-loaded PAMS decreased gradually.

The PAMS was used to remove Cu(II), Pb(II), Cd(II) and Cr(III) ions from aqueous solution. Compared with the results in the literature, the adsorption capacities with Langmuir fitting were higher and could be up to 2.33 mmol g^−1^ for Cu(II), 1.25 mmol g^−1^ for Pb(II), 0.83 mmol g^−1^ for Cd(II) and 0.56 mmol g^−1^ for Cr(III) at pH 5.0. The adsorption behaviour suggests that the adsorption was prompted by chelation interaction. Adsorption kinetics followed the pseudo second-order kinetic equation. Competition from common coexisting ions, such as Na(I), K(I), Ca(II) and Mg(II), was negligible, whereas the coexisting Fe(III) and Zn(II) ions displayed only a minor influence, which illustrated the selective adsorption of Pb(II), Cu(II) and Cr(III) from wastewater. Competitive adsorption among the four metal ions displayed a preferential adsorption of Cu(II) > Pb(II) > Cd(II) > Cr(III). The adsorption–desorption cycles demonstrated that the PAMS was suitable for reuse in the removal of heavy metal ions.

## Data accessibility

This article has no additional data.
